# Desmoglein 2 regulates the intestinal epithelial barrier via p38 mitogen-activated protein kinase

**DOI:** 10.1038/s41598-017-06713-y

**Published:** 2017-07-24

**Authors:** Hanna Ungewiß, Franziska Vielmuth, Shintaro T. Suzuki, Andreas Maiser, Hartmann Harz, Heinrich Leonhardt, Daniela Kugelmann, Nicolas Schlegel, Jens Waschke

**Affiliations:** 1 Department I, Institute of Anatomy and Cell Biology, Ludwig-Maximilians-Universität München, Pettenkoferstr. 11, 80336 Munich, Germany; 2 Department of Bioscience, School of Science and Technology, Kwansei Gakuin University, Sanda-shi, Hyogo-ken 669-1337 Japan; 3 Department of Biology II, Ludwig-Maximilians-Universität München, Großhaderner Str. 2, 82152 Planegg-Martinsried, Germany; 4 Department of General, Visceral, Vascular and Paediatric Surgery, Julius-Maximilians-Universität, Oberdürrbacher Str. 6, 97080 Würzburg, Germany

## Abstract

Intestinal epithelial barrier properties are maintained by a junctional complex consisting of tight junctions (TJ), adherens junctions (AJ) and desmosomes. Desmoglein 2 (Dsg2), an adhesion molecule of desmosomes and the only Dsg isoform expressed in enterocytes, is required for epithelial barrier properties and may contribute to barrier defects in Crohn’s disease. Here, we identified extradesmosomal Dsg2 on the surface of polarized enterocytes by Triton extraction, confocal microscopy, SIM and STED. Atomic force microscopy (AFM) revealed Dsg2-specific binding events along the cell border on the surface of enterocytes with a mean unbinding force of around 30pN. Binding events were blocked by an inhibitory antibody targeting Dsg2 which under same conditions activated p38MAPK but did not reduce cell cohesion. In enterocytes deficient for Dsg2, p38MAPK activity was reduced and both barrier integrity and reformation were impaired. Dsc2 rescue did not restore p38MAPK activity indicating that Dsg2 is required. Accordingly, direct activation of p38MAPK in Dsg2-deficient cells enhanced barrier reformation demonstrating that Dsg2-mediated activation of p38MAPK is crucial for barrier function. Collectively, our data show that Dsg2, beside its adhesion function, regulates intestinal barrier function via p38MAPK signalling. This is in contrast to keratinocytes and points towards tissue-specific signalling functions of desmosomal cadherins.

## Introduction

The internal surface of the gut is covered by a single layer of polarized enterocytes, forming the intestinal epithelium that operates as a selective barrier which protects the organism against luminal pathogens but allows uptake of nutrients. Barrier properties are established by three types of intercellular junctions, tight junctions (TJ), adherens junctions (AJ) and desmosomes which together form the “terminal bar” by sealing the paracellular space^1, 2^. TJ composed of claudins, occludin and a range of additional transmembrane proteins, are located in the most apical part where they seal the intercellular cleft^3^. Beneath, AJ formed by E-cadherin (E-cad) and a set of associated proteins mediate mechanical strength between epithelial cells and in addition are also involved in epithelial polarization, differentiation, migration and tissue morphogenesis^4^. The third and least studied type of intercellular junctions are the desmosomes, composed of the cadherin family members desmoglein (Dsg) and desmocollin (Dsc), which interact in homo- and heterophilic fashion via their extracellular domains (ED) and are associated with the intermediate filament cytoskeleton through specific desmosomal plaque proteins, namely plakoglobin (PG), plakophilins (Pkp) and desmoplakin (DP)^5^. Desmosomal cadherins are expressed as multiple isoforms in a tissue- and differentiation-specific manner. Layer specific expression pattern of all human isoforms (Dsg1-4 and Dsc1-3) can be observed in stratified epithelia such as the human epidermis whereas desmosomes in the simple columnar epithelium of the human intestine are composed of Dsg2 and Dsc2 only^6–9^.

Desmosomes are assumed to play the leading role in intercellular cohesion^10^. Beyond, they are also implicated in modulating fundamental cellular processes such as proliferation, apoptosis or organization of the cytoskeleton^11^. We have previously shown that desmosomal adhesion is required for intestinal epithelial barrier function^12^. The maintenance and turn-over of junctional complexes has to be regulated tightly during the rapid cell renewal of every 4–5 days in the intestinal epithelium^13^. On the other hand, increased intestinal permeability is associated with severe inflammatory disorders such as Crohn’s disease (CD)^14–17^. Especially, Dsg2 has already been shown to play a role in inflammation^18^ and in the pathogenesis of CD as it was strongly reduced in the mucosa of patients suffering from CD whereas the AJ molecule E-cadherin was unaffected^19^. Tumor necrosis factor-α (TNF-α), which is a central cytokine in CD, resulted in impaired barrier properties whereas a tandem peptide stabilizing desmosomal adhesion rescued barrier function. Interestingly, similar to TNF-α, a Dsg2-specific antibody targeting the ED of Dsg2 increased permeability^12^. However, it is unclear how this effect is achieved. It is likely that some amount of antibody permeates across TJs and directly inhibits transinteraction of Dsg2 within desmosomes, which compromises barrier integrity.

Another possibility could be that Dsg2 is expressed outside of desmosomes on the cell surface, accessible to the Dsg2-specific antibody and binding resulted in activation of signalling pathways. Desmogleins have already been shown to mediate signalling events^20^, however, nothing is known about extradesmosomal Dsg2 on the cell surface of enterocytes. In contrast, in keratinocytes extradesmosomal Dsg3 but not Dsg2 has been found in a signalling complex together with E-cadherin, β-catenin and Src^21^ where Dsg3 strengthens cell cohesion via modulation of mitogen-activated protein kinase (p38MAPK)^22^.

Bearing in mind that Dsg2 is the only Dsg isoform expressed in enterocytes and in view of our previous finding that it may contribute to the pathogenesis of CD, we investigated whether Dsg2 plays a role in modulating signalling cascades and cell cohesion in enterocytes, in this study. For the recent study, we used DLD1 cells deficient for Dsg2 and or Dsc2 under conditions where they were polarized similar to the well-established model of Caco2 cells used in our previous studies. Here, we show for the first time that extradesmosomal Dsg2 is expressed on the surface of polarized cultured enterocytes. Moreover, our data identify a novel role for Dsg2 in regulating p38MAPK as this kinase was activated after application of the Dsg2-specific antibody and reduced levels of p38MAPK activity were detected in Dsg2 knockout cells. Furthermore, activation as well as inhibition of p38MAPK led to barrier-destabilization, suggesting that a well-balanced level of p38MAPK activity is crucial for barrier properties. Collectively, our data provides evidence that Dsg2 regulates barrier properties in enterocytes via modulating the p38MAPK signalling cascade.

## Results

### Polarized cultured enterocytes displayed extradesmosomal Dsg2 at the cell surface

**Figure 1:**
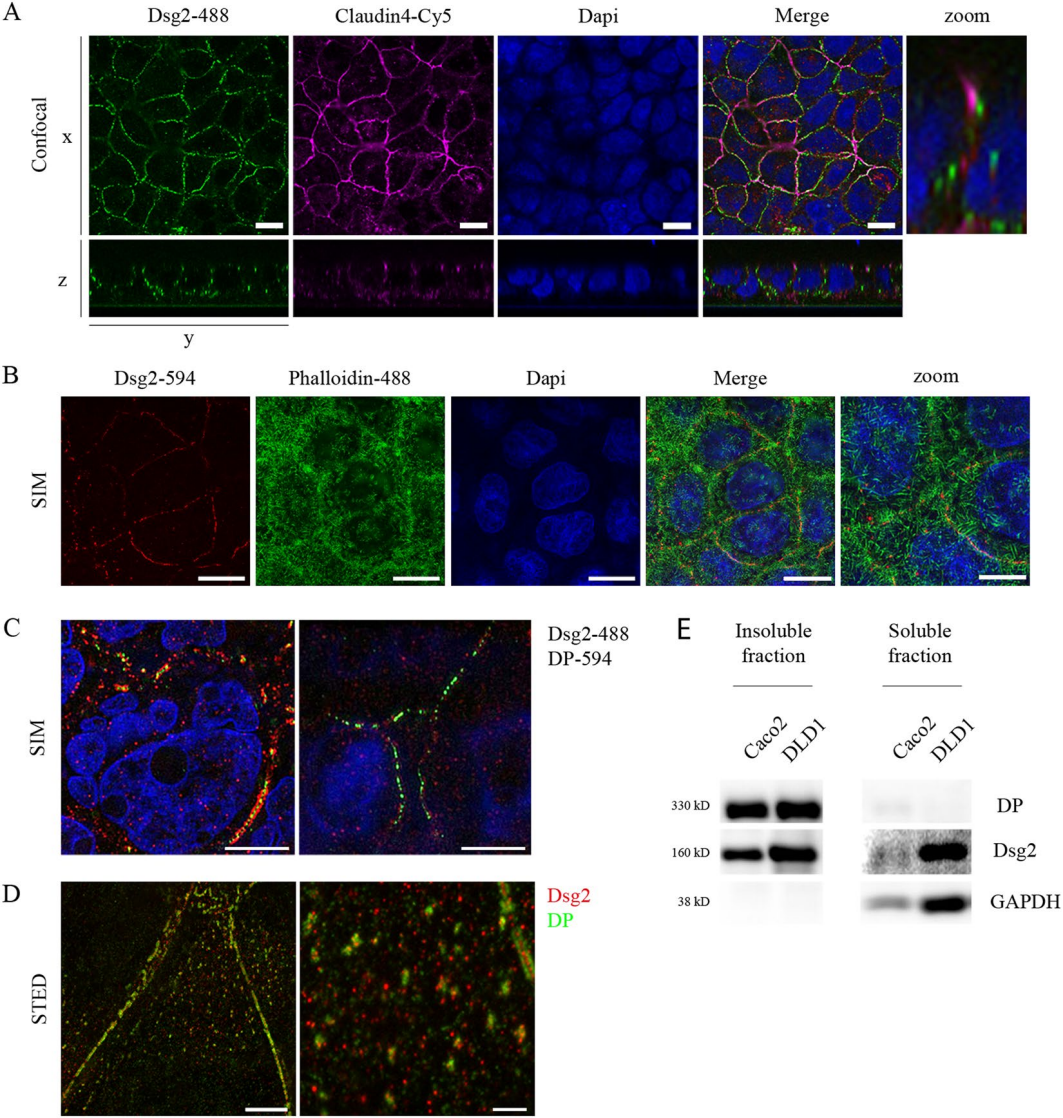
Extradesmosomal Dsg2 is present at the cell surface of polarized cultured enterocytes. Cells were grown on coverslips for several days after reaching confluency, fixed with 2% PFA and stained for junctional components. (**A**) Confocal microscopy analysis of Caco2 cells shows linear and apical localization of the junctional components Dsg2 and Cld4 at cell borders. Scale bar, 10 µm. (**B**) Analysis with SIM shows Dsg2 being located at same level as microvilli, visualized with Alexa488-phalloidin, at the surface of Caco2 cells. Shown is a Z-projection. Bar, 5 µm. (**C**) Apical fraction of Dsg2 (right panel) is not co-localizing with DP in contrast to lower layers (left panel) where both can be found in close proximity as analysed via SIM. Scale bar, 5 µm. (**D**) Both, clusters consisting of Dsg2 and DP as well as single Dsg2 molecules are present on the cell surface of DLD1 cells, as revealed by STED. Scale bar, 5 µm (left panel), 1 µm (right panel). (**E**) Dsg2 was detected in both, the Triton X-100-soluble and -insoluble fraction in contrast to DP being present only in the insoluble fraction. GAPDH served as loading control. Cropped blots are displayed and full-length blots are included in the supplementary information.

To explore the adhesive and signalling function of Dsg2 in the intestine, we used Caco2 and DLD1 cell lines. Both form an enterocyte-like epithelial cell monolayer with fully formed junctional complexes and characteristic microvilli on the cell surface (Fig. S1A) resembling a human specimen from the terminal ileum (Fig. S1B). Immunostaining of the junctional components Dsg2 and Claudin4 (Cld4) revealed linear localization at the cell border with junctional complexes being present at the most apical part of the intercellular cleft (Fig. 1A and S1C). Moreover, constant protein levels of junctional components (Fig. S1D) as well as constant transepithelial resistance (TER) values (Fig. S1E) revealed mature barrier properties. By using structured illumination microscopy (SIM), we observed that Dsg2 is also located at the free cell surface which is characterized by microvilli (Fig. 1B). Furthermore, SIM allowed distinguishing between desmosomal Dsg2 which is in close proximity to DP and extradesmosomal Dsg2 which is found solitary. Sandwich-like arrangement of Dsg2 and DP was observed at the lower lateral cell borders whereas apically, Dsg2 was found primarily separate from DP (Fig. 1C). Additionally, by using stimulated emission depletion microscopy (STED), we identified clusters on the cell surface consisting of Dsg2 and DP molecules as well as single Dsg2 molecules (Fig. 1D). These results were confirmed biochemically by triton extraction which separates the cell lysate into a soluble fraction and an insoluble fraction which is considered to be cytoskeleton-bound and to contain desmosomal components. In contrast to DP, which was detected in the insoluble fraction only, Dsg2 was found in both the insoluble as well as the soluble fraction (Fig. 1E) in line with extradesmosomal localization of Dsg2. Taking together, our findings reveal that Dsg2 is present outside of desmosomes on the cell surface in addition to its typical localization to serve as adhesion molecule in desmosomes.

### Dsg2-specific binding events were detected on the surface of living enterocytes

**Figure 2:**
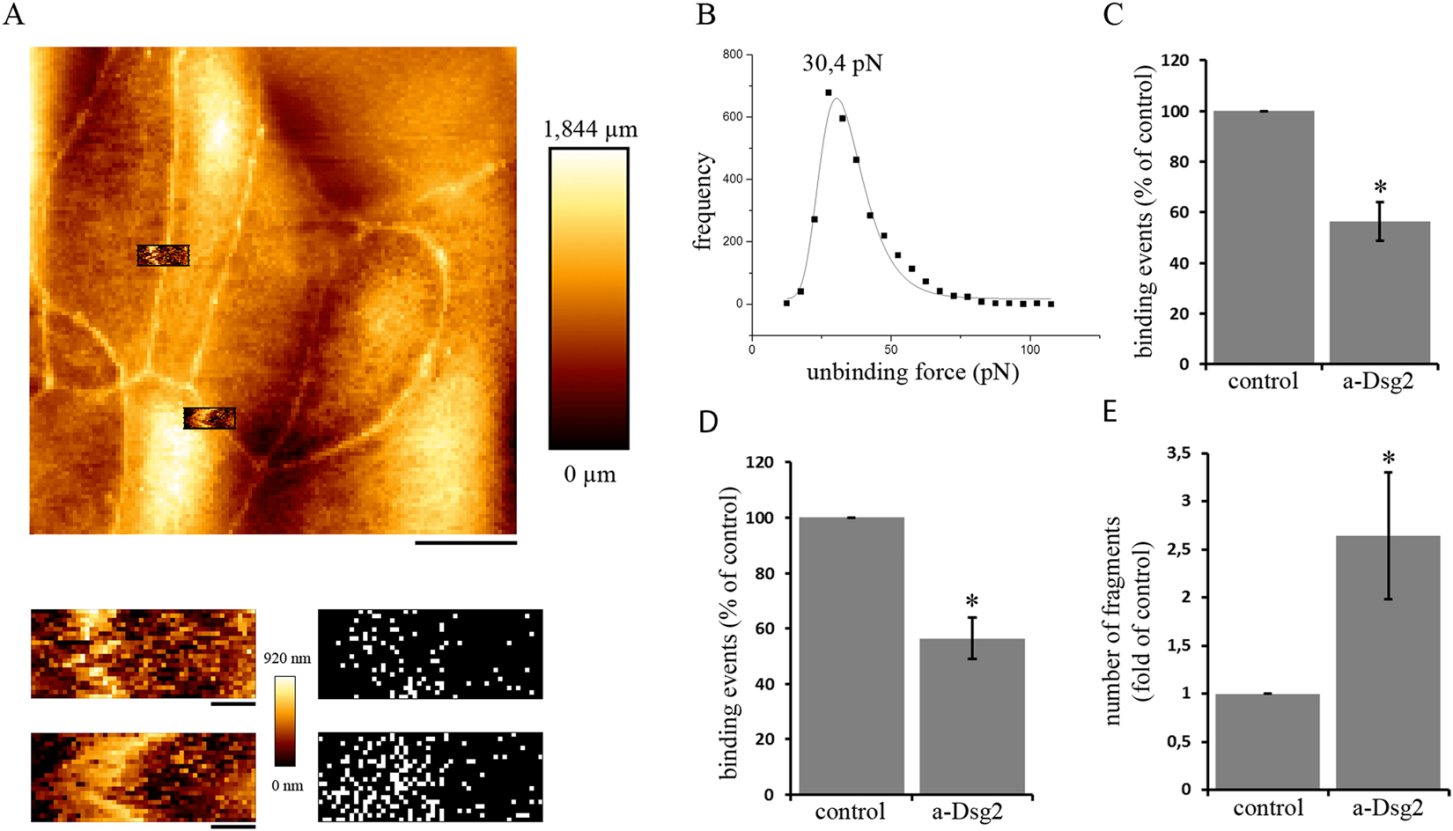
Dsg2-specific binding events can be detected on the surface of enterocytes. (**A**) Dsg2 force measurements were performed on living DLD1 cells at 37 °C. Cell topography was imaged to select specific areas at cell borders (upper panel). Force measurements revealed binding events along the cell border (lower panel) Bar, 10 µm (upper panel), 1 µm (lower panels). (**B**) Peak fit analysis of unbinding force resulted in a distribution-peak of 30,4 pN. (**C**) Application of a Dsg2-specific antibody significantly reduced the amount of binding events on living DLD1 cells as well as (**D**) in a cell-free setup (shown are means ± SE, n = 3, *p < 0,05). (**E**) Dsg2-specific antibody increased cell monolayer fragmentation in a dispase-based cell dissociation assay. (Shown is mean ± SE, n = 9, *p < 0,05 compared to control).

Since immunostaining revealed Dsg2-specific spots on the cell surface, we next applied AFM on living cells to characterize Dsg2-specific binding events similar as shown recently for Dsg3^23, 24^. The AFM topography image of DLD1 cells closely resembled the scanning electron microscopy (SEM) image of these cells (Fig. S2A) and allows specific measurements at cell borders which appeared elevated in the topography image. For each experiment, 2–3 areas at cell borders were selected for each condition, with 1000 recorded force-distance curves for each area. Under control conditions, binding events with a frequency of around 14,5% were detected which were more prominent close to the cell border (Fig. 2A). Peak fit analysis of unbinding force in DLD1 cells revealed a distribution-peak at 30,4 pN (Fig. 2B). To demonstrate specificity of binding events, an inhibitory Dsg2-specific antibody was added after control measurements^12, 25^. The antibody significantly reduced the binding frequency by around 40% (Fig. 1C). Specificity of the Dsg2-specific antibody was verified in a cell-free AFM setup, where it significantly blocked homophilic Dsg2 interaction to a similar extent (Fig. 2D). Similarly, siRNA-mediated silencing of Dsg2 expression resulted in significantly reduced binding frequency (Fig. S2B). Next, the antibody was applied in a dispase-based cell dissociation assay to investigate its effect on cell adhesion. Application for 24 h resulted in increased cell monolayer fragmentation compared to controls in both DLD1 and Caco2 monolayers (Fig. 2E and S2C). Depletion of Dsg2 using siRNA yielded similar results in both DLD1 and Caco2 cells (Fig. S2D). These observations further confirm the presence of extradesmosomal Dsg2 on the cell surface which specifically can be targeted by an anti Dsg2 antibody which is capable of reducing cell cohesion.

### Inhibition of Dsg2 binding activated p38MAPK which is critical for enterocyte cohesion

**Figure 3:**
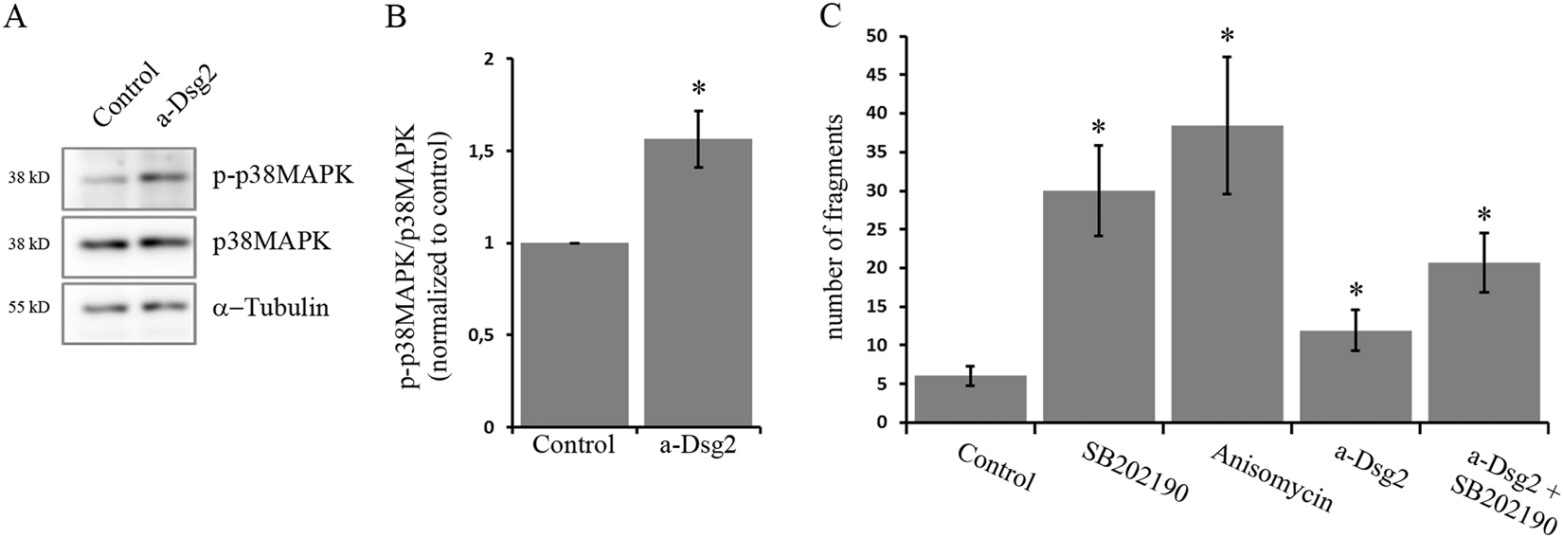
Inhibition of Dsg2 binding resulted in increased p38MAPK activity which is critical for enterocyte cohesion. (**A**) Western blot analysis after incubation of DLD1 cells with a Dsg2-specific antibody for 30 min revealed increased phosphorylation of p38MAPK. Cropped blots are displayed and full-length blots are included in the supplementary information. (**B**) Band intensity of detected p-p38MAPK was quantified using ImageJ and normalized to control (shown is mean ± SE, n = 6, *p < 0,05 compared to control). (**C**) DLD1 cells were treated with the p38MAPK inhibitor SB202190 or the activator anisomycin and analysed in a dispase-based cell dissociation assay. Both resulted in increased cell monolayer fragmentation. (Shown is mean ± SE, n = 3, *p < 0,05 compared to control).

In addition to their adhesive functions, desmosomal cadherins have already been shown to regulate p38MAPK signalling in keratinocytes^22, 26, 27^. Hence, we investigated the effect of inhibited Dsg2 binding on p38MAPK activity in enterocytes. After incubation with the Dsg2-specific antibody for 30 min, we observed activation of p38MAPK (Fig. 3A and B). Depletion of Dsg2 by siRNA did not cause p38MAPK activation but rather resulted in reduced level of p38MAPK activity (Fig. S3A), which is similar to DLD1 cells lacking Dsg2 (see below). Next, we examined whether the activity of p38MAPK is important for cell cohesion. Blocking of p38MAPK with the inhibitor SB202190 as well as activation of p38MAPK with anisomycin resulted in increased cell monolayer fragmentation (Fig. 3C). Moreover, application of the p38MAPK inhibitor together with the Dsg2-specific antibody also increased fragmentation (Fig. 3C). Intriguingly, short incubation with the Dsg2-specific antibody for 30 min was sufficient to activate p38MAPK but not to reduce cell cohesion (Fig. S3B), suggesting that binding to extradesmosomal Dsg2 may induce p38MAPK signalling. In contrast, incubation for 24 h increased cell monolayer fragmentation and was accompanied with elevated p38MAPK activity (Fig. S3C). Together, these data indicate that inhibition of Dsg2 binding activates p38MAPK which is critical for enterocyte cohesion and that the level of activated p38MAPK has to be well-balanced.

### Balance of p38MAPK activity is critical for intestinal barrier function

**Figure 4:**
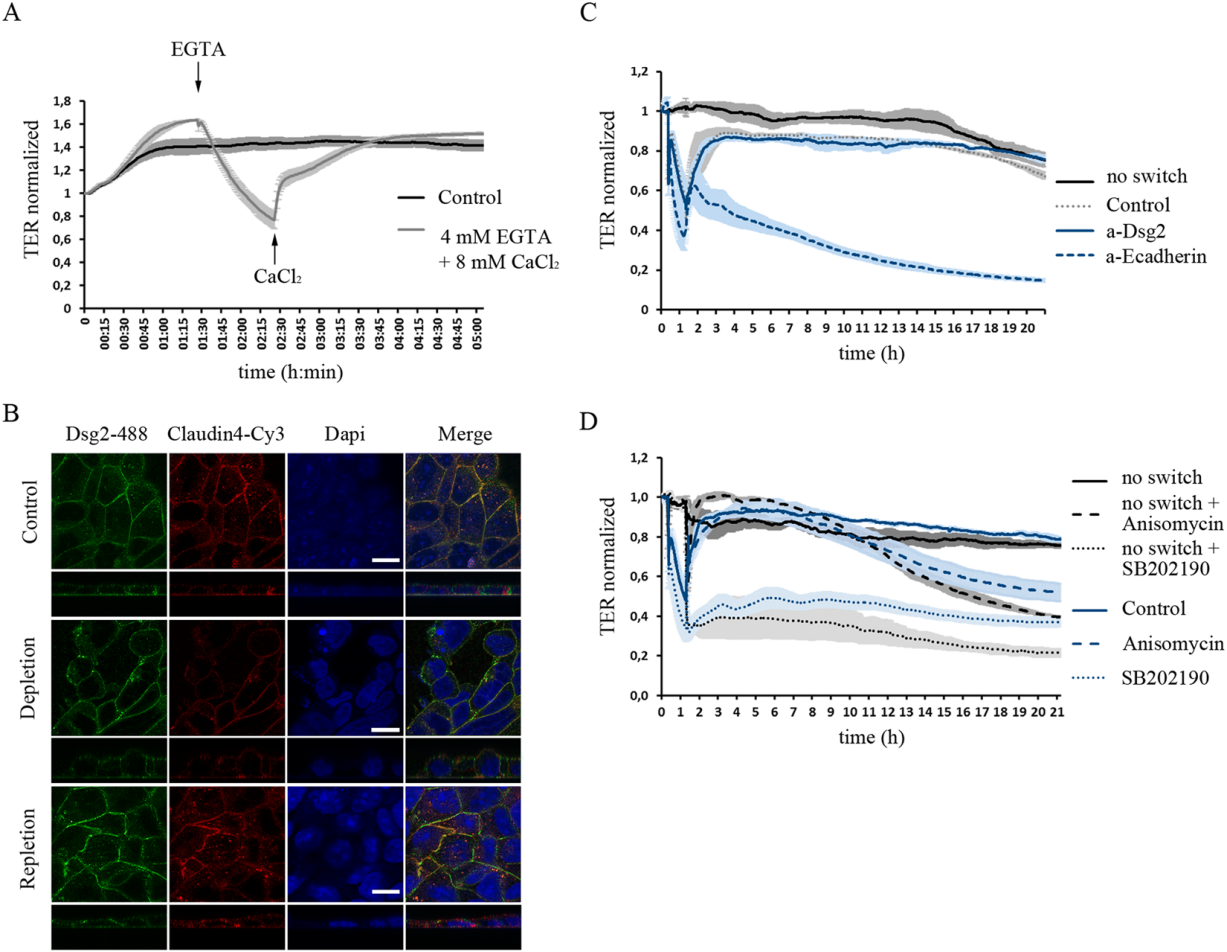
Well-balanced p38MAPK activity is critical for intestinal barrier function. Ca^2+^-switch assay with confluent DLD1 cells. (**A**) TER values decrease during depletion with 4 mM EGTA for 1 h and increase to back to control values during 2 h of repletion with 8 mM CaCl_2_. (**B**) Immunostaining of Dsg2 and Cld4 shows reduced and fragmented staining as well as gaps after 1 h depletion and similar staining to control condition after 2 h repletion. (**C**) Barrier reformation is not disturbed after application of a Dsg2-specific antibody but is impaired after addition of an inhibitory anti E-cadherin antibody. (**D**) TER values decrease after inhibition of p38MAPK with SB202190 and also barrier reformation is impaired in the Ca^2+^-switch experiment. Activation of p38MAPK via anisomycin has no effect in the short run but also induces reduction of TER values after about 10 h. (shown are representative graphs for at least three independent experiments).

We have previously shown that Dsg2 is required for maintenance of intestinal barrier function^12^. Therefore, we next sought to explore the role of Dsg2 during barrier recovery. To this end, we performed Ca^2+^-switch experiments which allowed us to induce barrier recovery under well-defined conditions^28^. TER values of confluent monolayers dropped during 1 h of EGTA-mediated Ca^2+^ depletion by around 50% and rose immediately after addition of CaCl_2_ (Fig. 4A and S4A). After about two hours of repletion, TER values reached again control levels indicating that reformation of junctional complexes is completed. In line with this, immunostaining of Dsg2, E-cad and Cld4 displayed fragmentation and reduction at cell borders during depletion accompanied with formation of intercellular gaps (Fig. 4B and S4B–D). After two hours of repletion, immunostaining was similar to controls (Fig. 4B and S4C). To examine the role of Dsg2 during barrier recovery, we next applied the Dsg2-specific antibody at the end of depletion together with CaCl_2_. Interestingly, barrier recovery was not disturbed (Fig. 4C). In contrast, application of an inhibitory E-cad-specific antibody abolished barrier recovery. Next, we applied SB202190 at the end of depletion which in DLD1 cells, prevented barrier reformation and moreover also resulted in barrier disruption without Ca^2+^-switch (Fig. 4D). Similarly, in Caco2 cells, inhibition of p38MAPK significantly delayed barrier recovery (Fig. S4E and S4F). Activation of p38MAPK via anisomycin had no immediate effect on barrier function and recovery (Fig. 4D). However, 10 h after application of anisomycin TER values dropped indicating that a well-balanced level of activated p38MAPK is crucial for barrier maintenance. In summary, our findings support an indispensable role of p38MAPK in barrier reformation and maintenance.

### Dsg2 regulates p38MAPK activity which is required for barrier properties

**Figure 5:**
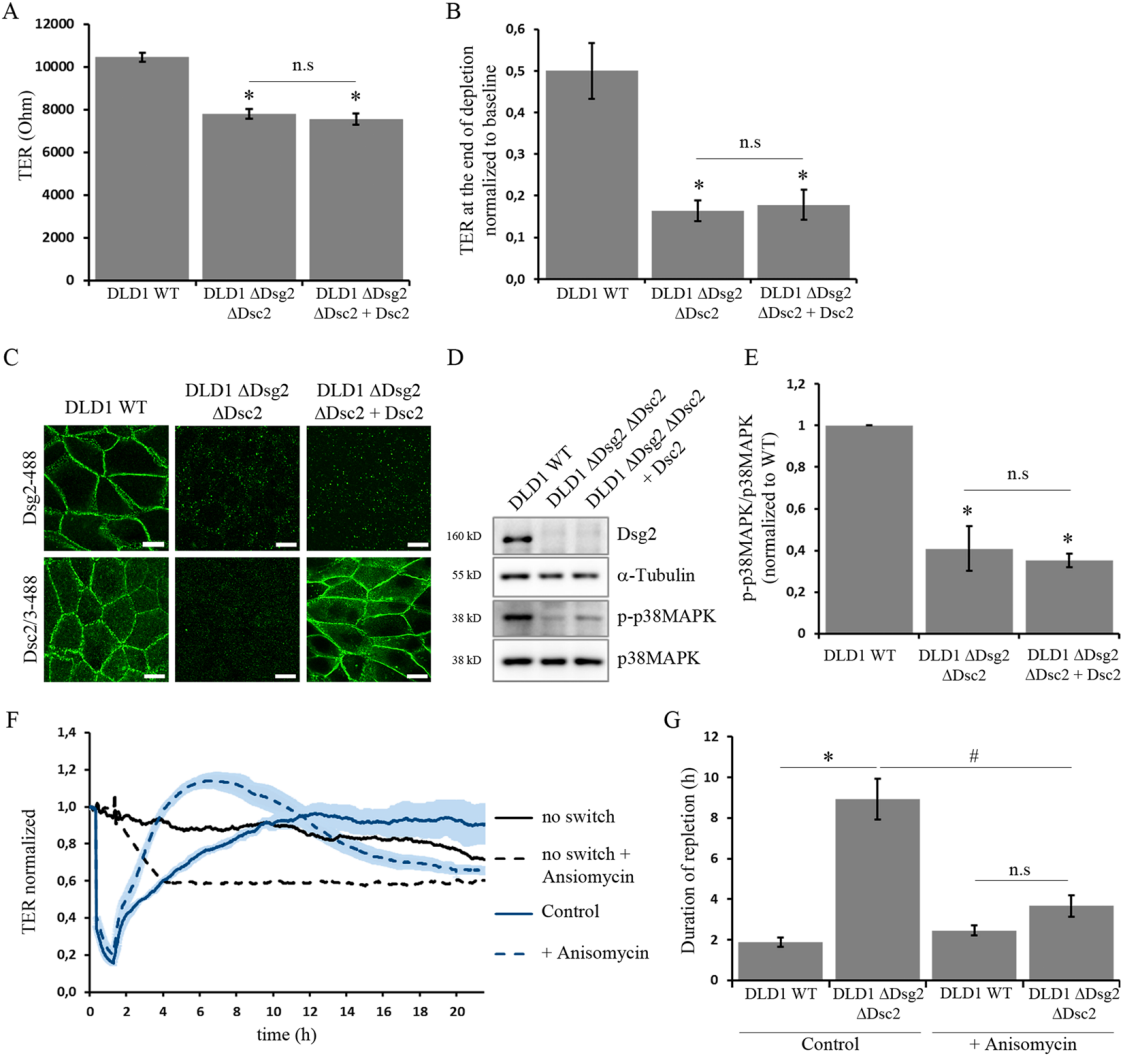
Dsg2 regulates p38MAPK activity which is required for barrier properties. (**A**) Comparison of baseline TER values between wildtype and knockout cells revealed reduced TER in DLD1 cells lacking Dsg2 (shown is mean ± SE, n ≥ 10, *p < 0,05 compared to control, n.s not significant). (**B**) During Ca^2+^ depletion for 1 h TER values of Dsg2-deficient cells decreased stronger compared to wildtype cells. (Shown is mean ± SE, n ≥ 6, *p < 0,05 compared to control, n.s not significant). (**C**) Wildtype and knockout cells grown on coverslips were stained for Dsg2 and Dsc2 to confirm knockout. Scale bar, 10 µm (**D**). Level of p-p38MAPK in Dsg2 and Dsc2 knockout cells was analysed via Western blot. Loss of Dsg2 and Dsc2 resulted in reduced level of p-p38MAPK which was not restored by Dsc2 rescue. α-Tubulin served as loading control. Cropped blots are displayed and full-length blots are included in the supplementary information. (**E**) Band intensity of detected p-p38MAPK was quantified using ImageJ and normalized to control (shown is mean ± SE, n ≥ 3, *p < 0,05 compared to control, n.s not significant). (**F**) Time for complete repletion during Ca^2+^-switch experiments was compared between wildtype and double knockout of Dsg2 and Dsc2. Loss of desmosomal cadherins resulted in a prolonged repletion time which was rescued by activation of p38MAPK via anisomycin (shown is mean ± SE, n ≥ 6, *p < 0,05 compared to wildtype under control conditions, #p < 0,05 compared to ΔDsg2ΔDsc2 under control conditions, n.s not significant). (**G**) Treatment with anisomycin reduced repletion time in ΔDsg2ΔDsc2 knockout cells. Representative graph for at least four independent Ca^2+^-switch experiments is shown.

Since the activation of p38MAPK via the Dsg2-specific antibody indicated that Dsg2 regulates p38MAPK activity, we hence determined alterations in p38MAPK signalling in DLD1 cells lacking desmosomal cadherins. Comparison of baseline TER values of wildtype (wt) and knockout cells revealed a significant reduction when desmosomal cadherins were absent (Fig. 5A). Interestingly, no difference was observed when both Dsg2 and Dsc2 were missing or when Dsc2 was re-expressed indicating that Dsg2 was critical for barrier properties. Similarly, in cells lacking Dsg2 chelation of Ca^2+^ had a more severe impact on cell junction disruption with values around 70% lower than wt values at the end of depletion (Fig. 5B). Interestingly, in both DLD1 cells missing Dsg2 and Dsc2 as well as in cells with Dsc2 rescue levels of p-p38MAPK but not total p38MAPK were reduced (Fig. 5D and E). This is similar to DLD1 cells where Dsg2 expression was suppressed by siRNA (Fig. S3A). This indicates that Dsg2 but not Dsc2 regulates the activity of p38MAPK. Finally, we investigated how the loss of desmosomal cadherins affects barrier recovery. Time for repletion was about 4–5 times higher in cells lacking Dsg2 and Dsc2 compared to wildtype (Fig. 5F and G). However, anisomycin to activate p38MAPK during Ca^2+^-switch experiments facilitated repletion in ΔDsg2ΔDsc2 cells resulting in times to recovery similar to wt cells (Fig. 5F and G). In summary, these data identify a new role for Dsg2 in regulating p38MAPK activity which is required for barrier properties in enterocytes.

## Discussion

In this study we demonstrate that Dsg2 is required for cell cohesion of enterocytes and maintenance of intestinal epithelial barrier function. For this function, regulation of p38MAPK appears to be critical, especially for barrier recovery. We detected reduced levels of activated p38MAPK in enterocytes deficient for Dsg2 and Dsc2 which was accompanied by both impaired barrier integrity as well as delayed barrier reformation. Reduced activity of p38MAPK was not restored with a Dsc2 rescue indicating that Dsg2 is required. However, barrier reformation was accelerated with direct activation of p38MAPK demonstrating that Dsg2-mediated activation of p38MAPK is crucial for barrier function. Furthermore, we identified extradesmosomal Dsg2 on the surface of polarized enterocytes. Application of an antibody targeting the ED of Dsg2 reduced binding events in AFM experiments and under same conditions activated p38MAPK but was not effective to reduce cell cohesion, indicating that extradesmosomal Dsg2 may be involved in the regulation of p38MAPK signalling.

In previous studies, we have characterized mechanisms regulating desmosomal adhesion in keratinocytes. However, desmosomal cadherins show tissue-specific expression patterns^6–9^, which implies that they may have distinct functionally properties within different tissues. In keratinocytes we found that Dsg2 is less important for cell cohesion compared to Dsg3^29^ and beyond that, activated p38MAPK reduces intercellular cohesion and forms a complex with Dsg3 but not Dsg2^22, 30^. In line with this, depletion of Dsg2 did not cause p38MAPK activation. In contrast, this study reveals that in enterocytes active p38MAPK is indispensable for barrier reformation and maintenance. Moreover, Dsg2 appears to regulate the activity of this kinase. However, by immunoprecipitation we did not find Dsg2 being associated with p38MAPK (data not shown).

Previously, we have shown that Dsg2 is required for intestinal barrier properties. Both, an antibody directed against Dsg2 as well as siRNA-mediated Dsg2 depletion resulted in loss of cell cohesion and reduced barrier function^12, 19, 22^. Since Dsg2 was missing at cell junctions in patients suffering from CD and a Dsg-specific tandem peptide ameliorated barrier dysfunction in response to TNF-α, which is regarded as a central cytokine in CD pathogenesis, we concluded that impaired Dsg2 may contribute to pathogenesis of inflammatory bowel diseases (IBD)^19^. This is supported by the finding that cytokines in IBD via matrix metalloproteinase 9 (MMP9) and a disintegrin and metalloproteinase domain-containing protein 10 (ADAM10) cause ectodomain cleavage of Dsg2, the products of which further compromised barrier integrity^18^. Thus, we were surprised that the Dsg2-specific antibody was not effective to inhibit barrier reformation in Ca^2+^-switch experiments, in contrast to an inhibitory E-cad-specific antibody which prevented barrier recovery completely. With this, it can be speculated that homophilic Dsc2 interactions can compensate for inhibited Dsg2 binding and are sufficient to restore barrier properties. Otherwise, it is also possible that AJ account for barrier establishment while desmosomes have rather regulatory function during epithelial homeostasis via p38MAPK. Studies from the literature showing that E-cad is clearly present at cell borders already at beginning of confluency^12^ support this hypothesis.

As outlined above, previous studies have shown that a specific antibody binding the ED of Dsg2 disrupted intestinal epithelial barrier properties^12^. Although, it is likely that this effect is to some extend caused by direct interference of Dsg2 binding within desmosomes, it is also possible that the Dsg2-specific antibody bound to extradesmosomal Dsg2 thereby inducing signalling events leading to reduced barrier properties. This is especially intriguing since extradesmosomal Dsg molecules have been proposed to serve as signalling hubs^31^. In this study, we demonstrated for the first time that Dsg2 is present outside of desmosomes on the surface of polarized cultured enterocytes. Using SIM and STED, we identified distinct Dsg2 spots on the cell surface as well as clusters consisting of Dsg2 and DP. Moreover, specific interaction between Dsg2-coated tips and the surface of living enterocytes was measured with AFM and application of the Dsg2-specific antibody resulted in decreased binding frequency, under conditions where it did not reduce cell cohesion in a dispase-based cell dissociation assay but significantly increased p38MAPK phosphorylation. Collectively, these findings suggest that the Dsg2-specific antibody binds to extradesmosomal Dsg2 on the cell surface thereby inducing p38MAPK signalling. However, incubation for 24 h reduced cell cohesion, indicating that it most likely interfered with desmosomal Dsg2 binding under these conditions. Along with a possible signalling function, Dsg2 is known to be a receptor for adenoviruses of which binding triggers intracellular signalling^32, 33^ also pointing towards the appearance of extradesmosomal Dsg2 on the cell surface as desmosomal Dsg2 is inaccessible for adenoviruses in polarized epithelial cells. Interestingly, Adenovirus 3 is supposed to bind to the ED 3 and 4 leading to an activation of members of the MAPK pathway^32, 33^. This is in accordance with our data, since the Dsg2-specific antibody is also directed against the ED 3 and induced p38MAPK activation, which was paralleled by reduced cell cohesion in a dispase-based cell dissociation assay. Considering that there is no enzymatic activity of the intracellular tail of Dsg2 known to date, the question arises how signalling cascades can be activated. A recent study demonstrated that soluble Dsg2 fragments activate the Akt/mTor and MAPK pathway through binding to HER2 and HER3 receptors^18^. In this line shedding of the Dsg2 ED upon binding to the virus was reported^32^, supporting the idea of Dsg2 fragments acting as ligands for other receptors. An alternative mechanism for activating signalling pathways may involve displacing proteins from lipid rafts as it has just been reported for Dsg2 in keratinocytes^34^. Furthermore, our data demonstrate that activity of p38MAPK has to be regulated tightly. Inhibition of p38MAPK impaired barrier reformation during Ca^2+^-switch experiments and augmented cell monolayer fragmentation. Direct activation of p38MAPK with anisomycin facilitated barrier recovery in Dsg2-deficient cells, in which baseline p38MAPK activity was reduced. However, in the course of several hours treatment with anisomycin impaired barrier function also. This is in line with our previous data, as treatment with TNFα activated p38MAPK which resulted in loss of cell cohesion and increased permeability as well as reduction of Dsg2 at the cell borders^19^.

Finally, our experiments also may give some insight into the pathogenesis of CD. As previously shown, Dsg2 is decreased in samples from patients suffering from CD^19^. However, it is unclear whether this is primary or secondary to lesion formation in CD and nothing is known about the underlying mechanisms leading to reduction of Dsg2. Our findings that extradesmosomal Dsg2 is present on the cell surface of enterocytes and Dsg2 modulates the p38MAPK signalling cascade could uncover a new mechanism in the development of CD. Dsg2 may act as a sensor to transmit extracellular stimuli thereby controlling intestinal barrier maintenance. In this scenario, it can be speculated that inappropriate transmission of environmental signals could lead to severe inflammatory responses and facilitate the development of CD. Further studies are necessary to prove this hypothesis.

## Materials and Methods

### Cell Culture

The two human intestinal epithelial cell lines Caco2 (ATTC, LGC Standards, Wesel, Germany) and DLD1 (kind gift of Shintaro T. Suzuki, Kwansei Gakuin University, Japan) were used. Caco2 cells were cultured in Eagle’s minimal essential medium (ATCC) and DLD1 cells were maintained in Dulbecco’s modified Eagle medium (Life Technologies, Carlsbad, CA) with both media being supplemented by 10% fetal bovine serum (Biochrom, Berlin, Germany), 50 U/ml penicillin and 50 U/ml streptomycin (both AppliChem, Darmstadt, Germany). Both cell lines were cultivated in a humidified atmosphere containing 5% CO_2_ at 37 °C and used for experiments after reaching full confluence.

### Test Reagents

SB202190 (Calbiochem, Darmstadt, Germany) was used to inhibit p38MAPK at 30 µM for 24 h and anisomycin (Sigma-Aldrich, Munich, Germany) was applied at 60 µM for 24 h to activate p38MAPK. For inhibition of Dsg2 binding a specific monoclonal mouse antibody directed against the second and third ED of Dsg2 (clone 10G11, Acris, Herford, Germany) without sodium azide was applied at 1:50. Following primary antibodies were used: mouse anti Dsg2 (clone 10G11) and rabbit anti Dsg2 (rb5, both Progen, Heidelberg, Germany), rabbit anti DP (NW6, USA, self-made), mouse anti Dsc2/3 (clone 7G6, Life Technologies, Carlsbad, CA), rabbit anti Claudin-4, rabbit anti Claudin-1 and rabbit anti Claudin-2 (all from Life Technologies, Carlsbad, CA), mouse anti E-cadherin (clone 36, BD Bioscience, Heidelberg, Germany,) mouse anti GAPDH (Santa Cruz Biotechnology, Santa Cruz, CA), mouse anti α-tubulin (Abcam, Cambridge, UK), rabbit anti p38MAPK, rabbit anti phospho-Thr180/182 p38MAPK, (Cell Signaling, Danvers, MA, USA),. As secondary antibodies HRP-conjugated goat anti-mouse or goat anti-rabbit (Dianova, Hamburg, Germany) were used for western blot analysis, Cy2-, Cy3- or Alexa488-labeled goat anti-mouse or goat anti-rabbit antibodies (Dianova, Hamburg, Germany) were used for confocal microscopy, Alexa488-labeled goat anti-mouse (Dianova, Hamburg, Germany) and Alexa594-labeled goat anti-rabbit (Life Technologies, Carlsbad, CA) were used for SIM and Star580- and Star635P-labeled antibodies (Abberior) were used for STED. For visualization of F-Actin Alexa488-labeled phalloidin (Life Technologies, Carlsbad, CA) was applied and nuclei were counterstained with DAPI (Sigma-Aldrich, Munich, Germany).

### Immunofluorescence

After grown to confluency on glass coverslips, cells were fixed with 2% paraformaldehyde in PBS for 10 min and subsequently permeabilized with 0,5% TritonX-100 in PBS containing 0,02% Tween20 (PBS-T) for 10 min. After blocking in 2% BSA in PBS-T for 1 h cells were incubated with primary antibodies for 1 h (all steps were performed at room temperature) and subsequently after 3 washing steps with PBS-T with secondary antibodies also for 1 h. For confocal microscopy coverslips were mounted on glass slides with 60% glycerol in PBS, containing 1,5% *N*-propyl gallate (Serva, Heidelberg, Germany) and images were acquired using a Leica SP5 confocal microscope with a 63 x NA 1.4 PL APO objective (both Leica, Wetzlar, Germany).

### Structured illumination microscopy (SIM)

After immunostaining, cells were mounted in VECTASHIELD (Vector Laboratories) and SIM images were acquired with a DeltaVisionOMX V3 microscope system (General Electric) equipped with a 100 × 1.4 oil immersion objective UPlanSApo (Olympus), 405 nm, 488 nm and 593 nm laser (DIC) and Cascade II camera (Photometrics). Reconstruction was done with the SoftWorx software (Vers. 6.0 Beta 19, unreleased) and additionally images were aligned with a self-written Fiji macro.

### Stimulated emission depletion microscopy (STED)

After immunostaining, cells were mounted in 2,5% DABCO in MOWIOL/HEPES (self-made solution) and imaged with an Abberior 3D STED confocal microscope. Star580 and Star635P (both from Abberior) were excited at 594 nm and 638 nm respectively using pulsed diode lasers (PDL 594, Abberior Instruments; PiL063X, Advanced Laser Diode Systems). Fluorescent molecules were depleted at 775 nm with a pulsed fibre laser (PFL-P-30-775B1R, MPB Communications) and emission was detected with an avalanche photodiode detector at 605-625 and 650-720 nm range.

### Western Blot

Cells were grown in 24-well plates and after treatment with respective reagents lysed using SDS lysis buffer containing (containing 25 mM HEPES, 2 mM EDTA, 25 mM NaF and 1% SDS) supplemented with a protease-inhibitor cocktail (Roche, Mannheim, Germany) and subsequently sonicated. After protein amount determination using a BCA Protein Assay Kit (Pierce/Thermo Scientific, Waltham, MA, USA) cell lysates were mixed with 3x Laemmli buffer and resolved by reducing SDS-PAGE. Following, proteins were transferred to a nitrocellulose membrane (Life Technologies, Carlsbad, CA) according to the standard protocols and membranes were probed with primary antibodies. Afterwards, HRP-conjugated secondary antibodies (Dianova, Hamburg, Germany) were applied and detected with an ECL reaction system (self-made solution).

### TritonX-100 Protein Extraction

Cell monolayer grown in 6-well plates were washed with ice cold PBS and incubated in a Triton buffer (containing 0.5% Triton X-100, 50 mM MES, 25 mM EGTA and 5 mM MgCl_2_) for 15 min on ice under gentle shaking. To separate the cytoskeletal protein fraction (Triton-insoluble) from the non-cytoskeletal protein fraction (Triton-soluble), cell lysates were centrifuged at 13,000 rpm for 5 min. After resuspending the pellets in SDS lysis buffer followed by sonication, protein concentration of both fractions was defined as described above and lysates were analysed via Western blot.

### Atomic Force Microscopy (AFM)

Dsg2 interactions on the surface of living enterocytes were analysed with atomic force microscopy (AFM), using a Nanowizard III AFM (JPK Instruments, Berlin, Germany) mounted on an optical microscopy (Carl Zeiss, Jena, Germany). The principle of AFM force spectroscopy was described in detail before^23^. Briefly, the sharp tip on a flexible cantilever was functionalized with recombinant Dsg2-Fc containing the complete ED of Dsg2 using a flexible polyethylene glycol linker (Gruber Lab, Institute of Biophysics, Linz, Austria) as outlined elsewhere^35^. While measurements, the tip is repetitively lowered onto the enterocyte cell monolayer and retracted again. Thereby binding events between the molecule on the tip and a molecule on the cell surface can be detected as the tip is hold back near the cell surface during the retraction movement in case of interaction. When the retraction force overcomes the binding strength of the interaction the tip jumps back into neutral position. AFM topography images of 50 × 50 µm were acquired at the beginning of each experiment in a force curve-based imaging mode (QI- mode) with a setpoint adjusted to 0,5 nN, a z-length of 1500 nm and a pulling speed of 50 µm/s. For force mapping to detect specific binding events a small area of 2 × 5 µm was chosen and a map consisting of 20 × 50 pixels with each pixel representing one force-distance curve was recorded. Hereby, relative setpoint was set to 0,5 nN, a z-length of 2 µm was used for the DLD1 cells and 3 µm for the Caco2 cells with a pulling speed of 4 µm/s for the DLD1 cells and 5 µm/s for Caco2 cells. Measurements were performed in respective cell culture medium at 37 °C. For cell-free AFM measurements Dsg2 coated mica sheets (SPI Supplies, West Chester, USA) were used instead of cell monolayers. Setpoint was adjusted to the same value as for cell-based experiments to produce comparable results. The z-length was set to 0,3 µm and a pulling speed of 1 µm/s was used. For each condition 400 force-distance curves were measured on an area of 25 µm × 25 µm.

### Dispase-based Dissociation Assay

Confluent Caco2 and DLD1 cell monolayer grown in 24-well plates were incubated with test reagents, washed with Hank’s buffered saline solution (HBSS; Sigma-Aldrich) and treated with 150 µl of the enzyme dispase II (2,4 U/ml in HBSS, Sigma-Aldrich) at 37 °C to detach the whole cell monolayer from the well bottom. For Caco2 cells additionally 1% Collagenase I (Thermo Scientific, Waltham, MA, USA) was added. DLD1 cell monolayer detached from the well bottom after 30 min and Caco2 cell monolayer after 1,5 h. After stopping the reaction with 200 µl HBSS cell monolayer were sheared by pipetting 3 times using an electrical pipet. Finally, resulting fragments were counted under a binocular microscope (Leica). For every condition 3–4 wells out of a 24-well plate were counted and each experiment was repeated at least 3 times.

### Measurement of transepithelial resistance

To asses epithelial barrier integrity, the transepithelial resistance (TER) was measured with an ECIS model Z theta (Applied Biophysics, Troy, NY) as described previously^36^. For this, cells were cultured on 8-well electrode arrays (Ibidi, 8W10E) with sensing areas consisting of working electrodes at each well bottom and a counter electrode. Via the electrolytes of the cell culture medium above the cells, the electrodes are electrically connected. By application of a non-invasive alternating current (<1 µA) to the electrodes, the ECIS device measures the associated voltage drop across the system and determines the electrical resistance of the cell covered electrodes. A drop in TER values mirrors barrier breakdown. At the beginning of measurements medium was exchanged (300 µl) and baseline TER at 400 Hz for Caco2 cells and 800 Hz for DLD1 cells, was measured until values reached a plateau. Then, different test reagents were added and resistance was monitored every minute for the next 24 h.

### Calcium Switch Assay

To analyse cell junctional disassembly and reassembly, Caco2 and DLD1 cells were grown on 8-well electrode arrays (Ibidi, 8W10E) and TER was monitored every minute. After measurement of baseline TER for about 20 min, Ca^2+^ ions were depleted using 4 mM EGTA for 1 h thereby inducing disruption of Ca^2+^-dependent cell junctions. With the addition of 8 mM CaCl_2_, reformation of cell junctions was induced and validated based on changes in TER values.

### Statistics

All experiments were repeated at least 3 times. Quantification of band intensity was performed using ImageJ (National Institutes of Health, Bethesda, MD). Statistical analysis was performed using a two-tailed *t* test for 2-sample group analysis and one-way ANOVA followed by bonferroni correction for multiple sample groups.

### Human samples

Human specimens were obtained from terminal ileum of patients that required right hemicolectomy in which the surgical resection routinely involves a part of the healthy small intestine. All experiments were performed in accordance with relevant guidelines and regulations. All patients had given their informed consent before surgery, and the study was approved by the Ethical Board of the University of Würzburg (proposal numbers 113/13 and 46/11). For TEM analyses samples were fixed with 2.5% glutaraldehyde, cut into ~1mm³ pieces and further processed as described above.

### Data Availability

The datasets generated during and/or analysed during the current study are available from the corresponding author on reasonable request.

## Electronic supplementary material

Supplementary information
