# Supplementary material for: Desmoglein 2 regulates the intestinal epithelial barrier via p38 mitogen-activated protein kinase
Source: Sci Rep. 2017 Jul 24;7:6329. doi: 10.1038/s41598-017-06713-y (PMC5524837; doi:10.1038/s41598-017-06713-y)
Supplement: Supplementary file 1 — Supplementary information [file 41598_2017_6713_MOESM1_ESM.pdf]

Supplementary material to the manuscript

Title: Desmoglein 2 regulates the intestinal epithelial barrier via p38 mitogen-activated protein kinase

Authors: Hanna Ungewiß[1], Franziska Vielmuth[1], Shintaro T. Suzuki[2], Andreas Maiser[3], Hartmann Harz[3], Heinrich Leonhardt[3], Daniela Kugelmann[1], Nicolas Schlegel[4] , Jens Waschke[1]*

Author affiliation:

[1] Department I, Institute of Anatomy and Cell Biology, Ludwig-Maximilians-Universität München, Pettenkoferstr. 11, 80336 Munich, Germany

[2] Department of Bioscience, School of Science and Technology, Kwansei Gakuin University, Sanda-shi, Hyogo-ken 669-1337, Japan

[3] Department of Biology II, Ludwig-Maximilians-Universität München, Großhaderner Str. 2, 82152 Planegg-Martinsried, Germany

[4] Department of General, Visceral, Vascular and Paediatric Surgery, Julius-Maximilians-Universität, Oberdürrbacher Str. 6, 97080 Würzburg, Germany

Supplemental Material and Methods

Transmission Electron Microscopy (TEM)

Cell monolayer were fixed with 1% glutaraldehyde at 37°C for 1 h, then washed three times with PBS and incubated with 2% osmiumtetroxid solution for 1 h at 4°C. Afterwards, samples were dehydrated through an ethanol series from 20 to 100% followed by embedding with epon for 24 hours at 80°C. Finally, ultrathin sections (60 - 80 nm) were cut with a diamond knife and staining was performed with a saturated solution of uranyl acetate for 40 min and lead citrate for 5 min. Images were acquired with the transmission electron microscope Libra 120 (Zeiss, Oberkochen, Germany).

Scanning Electron Microscopy (SEM)

For scanning electron microscopy, cell monolayer were fixed and dehydrated as described for TEM. Afterwards, samples were dried with carbon dioxide under excess pressure of around 86 bar (Critical point dryer K850, Quorum Technologies, UK) and subsequently sputtered with gold at 30 mA for 60 s (Cressington 108auto) under argon atmosphere. Images were acquired using the scanning electron microscope Leo 1550 (Zeiss Oberkochen, Germany).

siRNA-mediated Silencing of Dsg2

Human siRNA oligo pools specific for Dsg2 as well as non-target controls were purchased from Dharmacon/GE Healthcare (Freiburg, Germany). Cells were transfected at 90% confluency using Turbofect (Thermo Scientific, Waltham, MA) as a transfection reagent according to manufacturer's protocols. Medium was exchanged after 24 hours and experiments were performed after 48 hours.

Supplementary figure legends

Figure S1. Caco2 cells were cultured for 2 days, 4 days or 6 days after confluency and analysed via transmission and scanning electron microscopy, confocal microscopy, Western blot and TER. (A) Characteristic junctional complexes as well as microvilli were present already on day 2. Scale bar, 1 µm (left and central panels), 2 µm (right panel) (B) Electron microscopy images of human terminal ileum specimens show tight junctions (TJ), adherens junctions (AJ) and desmosomes (D) at the apical

part of the intercellular cleft. Desmosomes can also be found in lower regions of the intercellular cleft. Scale bar, 1000 nm (left panel), 500 nm (right panel) (C) Immunostaining of junctional components reveals linear localization of Dsg2, Cld1 and Cld4 already on day 2, staining for Cld2 decreases in the time course of differentiation. Scale bar, 10 µm. (D) Western blot analysis reveals unaltered protein level of junctional components from day 2 till day 6. α-Tubulin serves as loading control. (E) TER measurements reveal constant values between day 2 and day 6. (n = 4, n.s not significant)

Figure S2. (A) AFM topography image and scanning microscopy image of DLD1 cells closely resemble each other. Scale bar, 2 µm (left panel), 10 µm (right panel). (B) siRNA-mediated silencing of Dsg2 reduces amount of binding events on living DLD1 cells. (siNT = non-target siRNA; shown is mean ± SE, n = 4, * p < 0,05) (C) Dsg2-specific antibody increases cell monolayer fragmentation of Caco2 cells in a dispase-based cell dissociation assay (shown is mean ± SE, n = 4, * p < 0,05). (D) siRNA-mediated silencing of Dsg2 increases cell monolayer fragmentation of DLD1 and Caco2 cells in a dispase-based cell dissociation assay. (shown is mean ± SE, n = 4, * p < 0,05)

Figure S3. (A) siRNA mediated silencing of Dsg2 reduces phosphorylation of p38MAPK. (B) Dsg2-specific antibody did not increase cell monolayer fragmentation in a dispase-based cell dissociation assay after 30 min of incubation (shown is mean ± SE, n = 4, n.s = not significant compared to control). (C) Western blot analysis after incubation of DLD1 cells with a Dsg2-specific antibody for 24 h revealed increased phosphorylation of p38MAPK. Band intensity of detected p-p38MAPK was quantified using ImageJ and normalized to control (shown is mean ± SE, n = 3, * p < 0,05 compared to control).

Figure S4. (A) TER values of Caco2 cells decrease during depletion with 4 mM EGTA for 1 h and increase back to control values during 2 h of repletion with 8 mM $CaCl_2$. (Representative graph for n > 3 is shown). (B) Immunostaining of Dsg2 and E-cadherin during Ca^{2+}-switch experiments in DLD1 cells reveals reduction and fragmentation after 1 h depletion and similar staining to control condition after 2 h repletion. Scale bar, 10 µm. (C-D) Immunostaining of junctional components during Ca^{2+}-switch experiments in Caco2 cells reveals reduction and fragmentation as well as gap formation after 1 h depletion and similar staining to control condition after 2 h repletion. Scale bar, 10 µm. (E) Barrier

reformation in Caco2 cells is impaired after inhibition of p38MAPK with SB202190 in the Ca^{2+}-switch experiment while addition of anisomycin has no effect (Representative graph for 6 independent experiments is shown). (D) Time for repletion during Ca^{2+}-switch experiments was compared between control condition and application of anisomycin or SB202190. Inhibition of p38MAPK with SB202190 significantly delayed the repletion time while application of anisomycin resulted in a repletion time similar to control condition (shown is mean ± SE, n=6, * $p < 0,05$ compared to control condition, n.s not significant).

Supplemental Figure 1

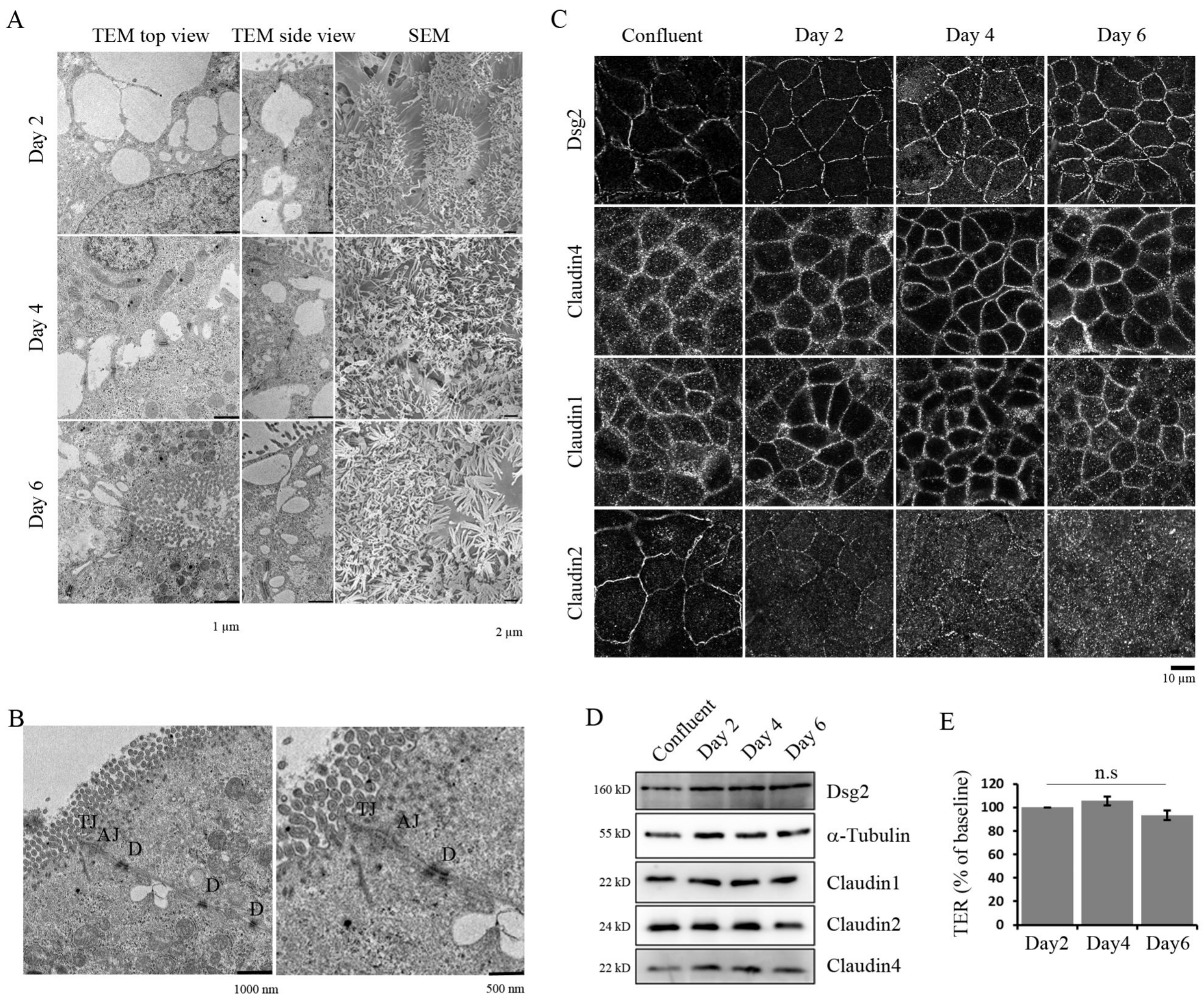

Supplemental Figure 2

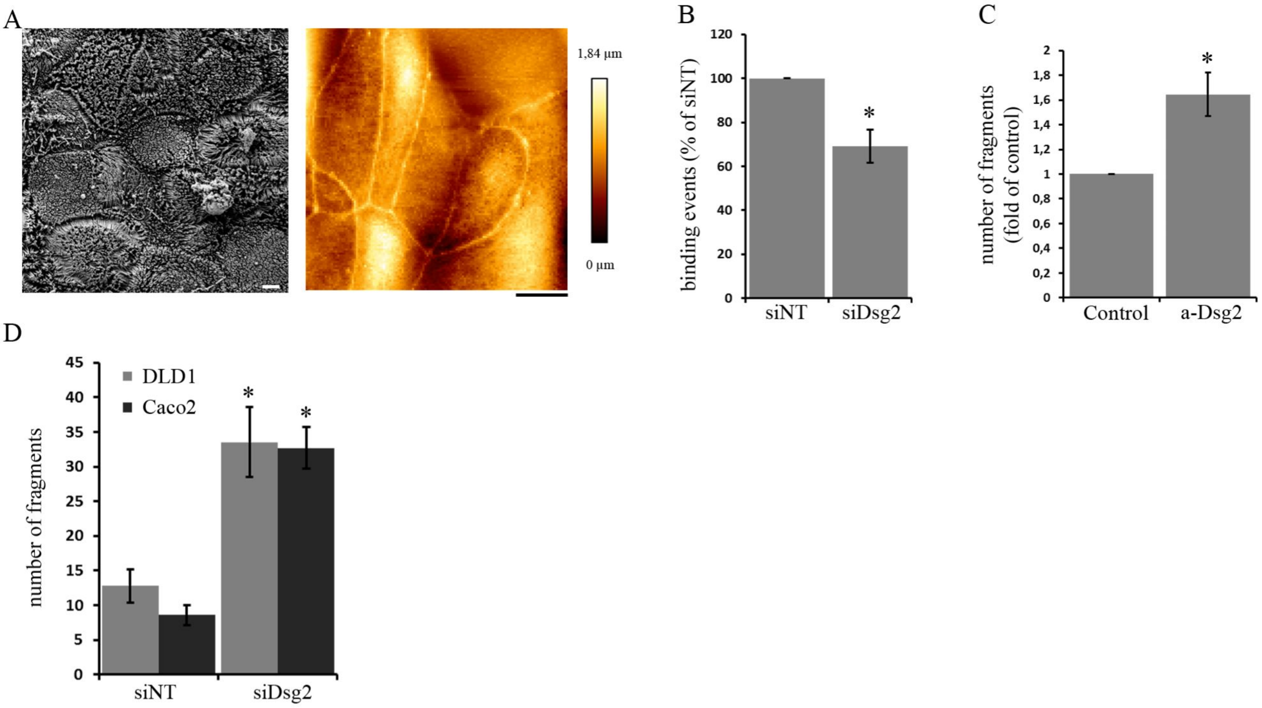

Supplemental Figure 3

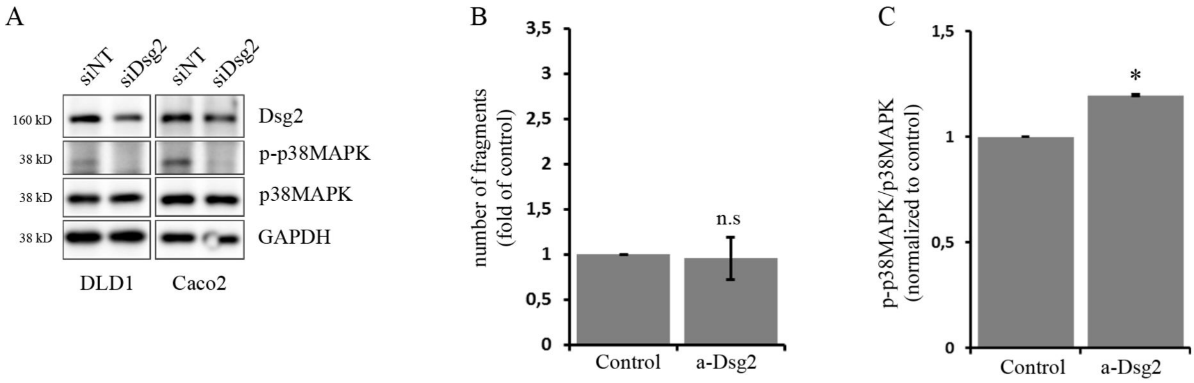

Supplemental Figure 4

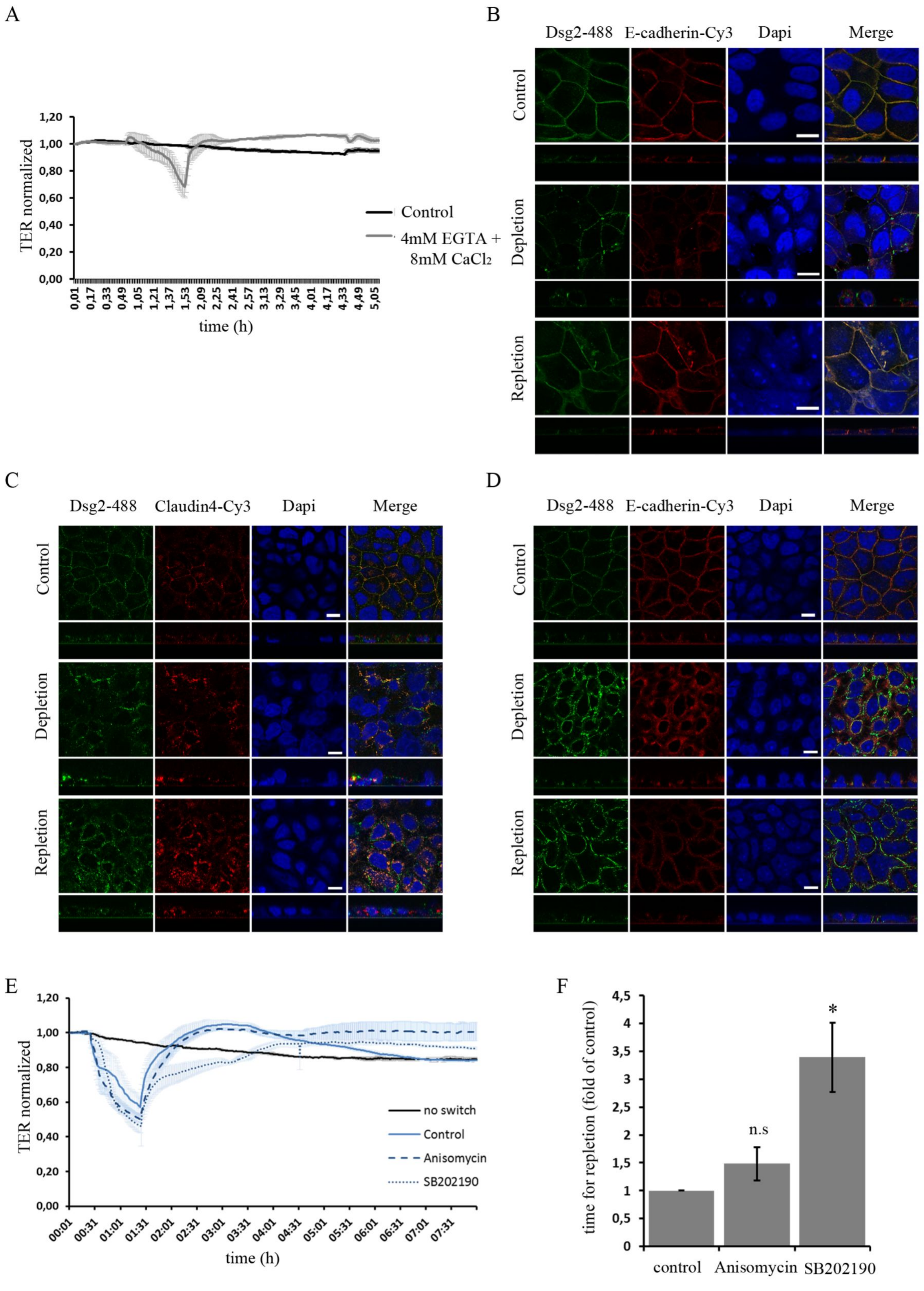

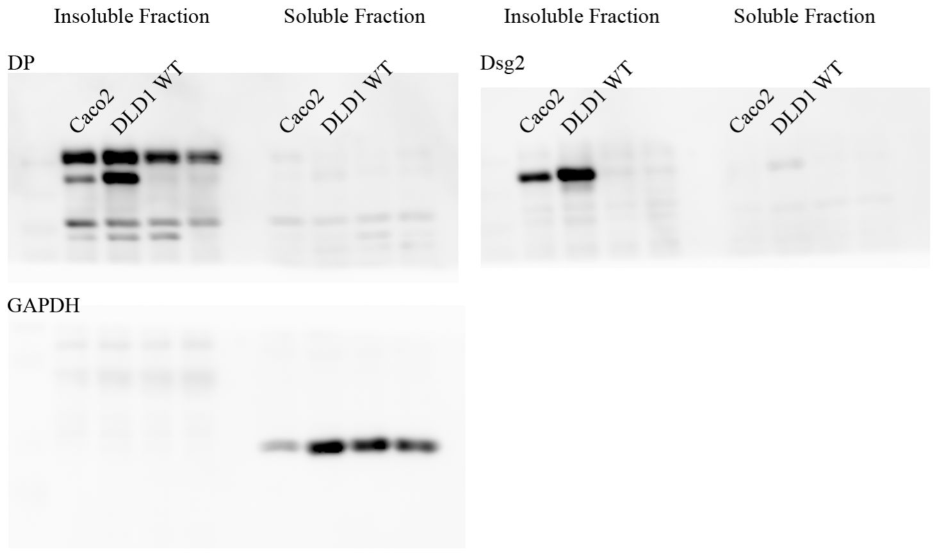

Full length blot for Desmoplakin, Desmoglein 2 and GAPDH displayed in figure 1E

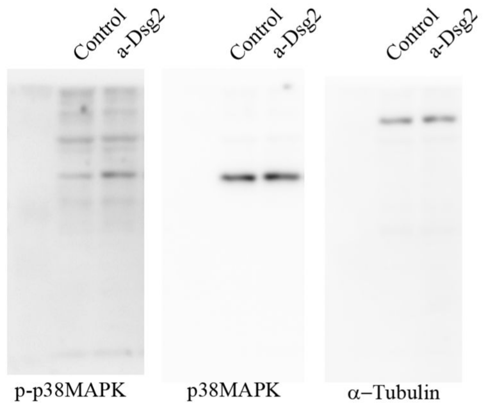

Full length blot for p-p38MAPK, p38MAPK and α−Tubulin displayed in figure 3A

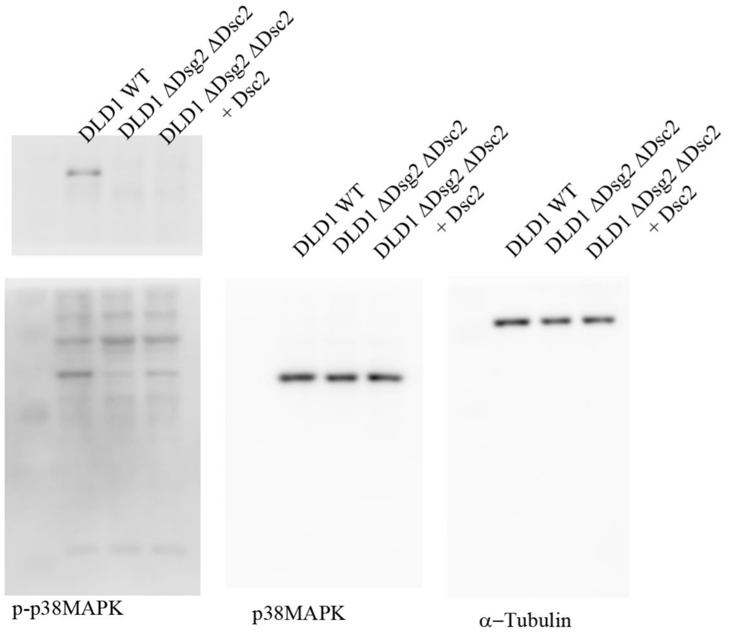

Full length blot for Dsg2, p-p38MAPK, p38MAPK and α−Tubulin displayed in figure 5D
